# Applicability of Mechanical Tests for Biomass Pellet Characterisation for Bioenergy Applications

**DOI:** 10.3390/ma11081329

**Published:** 2018-07-31

**Authors:** Orla Williams, Simon Taylor, Edward Lester, Sam Kingman, Donald Giddings, Carol Eastwick

**Affiliations:** Faculty of Engineering, The University of Nottingham, University Park, Nottingham NG7 2RD, UK; taylor_simon@hotmail.co.uk (S.T.); edward.lester@nottingham.ac.uk (E.L.); sam.kingman@nottingham.ac.uk (S.K.); donald.giddings@nottingham.ac.uk (D.G.); carol.eastwick@nottingham.ac.uk (C.E.)

**Keywords:** mechanical strength, biomass pellets, Split Hopkinson pressure bar, Instron mechanical tester, pellet durability, milling energy

## Abstract

In this paper, the applicability of mechanical tests for biomass pellet characterisation was investigated. Pellet durability, quasi-static (low strain rate), and dynamic (high strain rate) mechanical tests were applied to mixed wood, eucalyptus, sunflower, miscanthus, and steam exploded and microwaved pellets, and compared to their Hardgrove Grindability Index (HGI), and milling energies for knife and ring-roller mills. The dynamic mechanical response of biomass pellets was obtained using a novel application of the Split Hopkinson pressure bar. Similar mechanical properties were obtained for all pellets, apart from steam-exploded pellets, which were significantly higher. The quasi-static rigidity (Young’s modulus) was highest in the axial orientation and lowest in flexure. The dynamic mechanical strength and rigidity were highest in the diametral orientation. Pellet strength was found to be greater at high strain rates. The diametral Young’s Modulus was virtually identical at low and high strain rates for eucalyptus, mixed wood, sunflower, and microwave pellets, while the axial Young’s Modulus was lower at high strain rates. Correlations were derived between the milling energy in knife and ring roller mills for pellet durability, and quasi-static and dynamic pellet strength. Pellet durability and diametral quasi-static strain was correlated with HGI. In summary, pellet durability and mechanical tests at low and high strain rates can provide an indication of how a pellet will break down in a mill.

## 1. Introduction

The safe and effective transportation and processing of biomass pellets is critical for bioenergy applications [1]. The integrity of the pellets during transport and fuel handling is key in ensuring a standardised product for processing [2], and it is essential that pellets do not degrade physically between manufacture and use. This minimises transport costs and reduces the risk of fires through dust explosions [3]. There have been numerous studies into the chemical properties of biomass [4]. However, studies on the compressive mechanical strength of commercially produced biomass pellets [5], and how this relates to bioenergy storage, transport, and milling processes [6,7], are more disparate. This study explores the applicability of durability, and quasi-static and dynamic mechanical strength tests for biomass pellets for bioenergy applications. 

Low pellet mechanical resistance leads to high dust emissions; system blockages; and an increased risk of fire and explosions during pellet handling, storage, and transport [3]. Pellet durability is a standard test that measures the resistance of densified fuels to shocks and/or abrasion as a consequence of handling and transportation processes [8]. Minimum pellet durability requirements are detailed in BS EN ISO 17225-2 [9] and BS EN ISO 17225-6 [10]. Pellet durability is related to the effectiveness of densification [11], and can be affected by the pre-pelleting factors, such as feedstock characteristics [12,13], age and storage conditions of the raw material [14], moisture content [15,16], and processing conditions (binders, feedstock mixes, temperatures, or die pressures) [1,17,18]. Additionally, storage [5,6,19] and handling frequency [17] impacts durability. Pre-treatments, such as steam explosion, have been shown to improve wood pellet density, durability, and impact resistance [7,20]. Pellet durability has been shown to correlate to lab milling energy for a laboratory scale knife mill [21]. However, no other studies have explored this correlation.

The quasi-static mechanical strength of pellets is the compressive strength of pellets at low to moderate strain rates (10^−4^–10^0^ s^−1^) [22]. The compressive strength of biomass pellets has been assessed using a wide range of compressive resistance tests, including the universal materials testing machine [23]. However, repeatable results are difficult to obtain because of the heterogeneous nature of biomass. Compressive strength is known to depend on the material composition, densification process, and final physical properties of the pellet [24,25,26]. Most compressive strength studies have been conducted on pellets produced in single pellet dies [16,24,26,27,28,29,30,31] or laboratory pilot scale systems [32,33,34], with only a few studies on commercially produced pellets [5,6,7,35]. Smaller pellet particle size [30,32] and torrefaction [31,35] are known to improve the mechanical strength of pellets, while long-term storage has been shown to reduce mechanical strength [5,6]. Larsson et al. [34] found that compressive strength cannot be considered as a reliable indirect measure for ISO 17831-1:2015 standard fuel pellet durability [8]. Shang et al. [7] found a correlation between diametral compressive strength and milling energy in a commercial coffee grinder for torrefied Scots pine pellets. Yet to date, no studies have related the mechanical properties of different species of biomass pellets and milling energy.

The dynamic mechanical strength of a material is a measure of its strength at high strain rates (10^2^–10^4^ s^−1^) [36]. The tests aim to determine the material properties under hammer-blowing conditions, which are similar to those experienced with comminution systems [37]. A controlled method of analysing these mechanical properties is through a Spilt Hopkinson pressure bar (SHPB) [38]. The SHPB test has been used to analyse the forces within mills [39], and analyse the strength of cellulosic materials [40]. Ultrafast load cells (UFLC) have been used to analyse the fracture of single particles under impact loading [39,41], but these tests involve point loads rather than uniformly distributed loads. To date, the dynamic mechanical strength of biomass pellets has yet to be explored in literature. This paper presents an assessment of pellet durability, quasi-static mechanical strength, and a novel analysis of the dynamic mechanical strength of several varieties of biomass pellets. The paper establishes potential correlations between mechanical properties and milling energies and evaluates the appropriateness of the tests for characterising biomass pellets during storage, transport, and comminution.

## 2. Materials and Methods

### 2.1. Materials

Six commercially sourced biomass pellets were evaluated. Portuguese mixed wood pellets (mainly pine (*Pinus*) with eucalyptus (*Eucalyptus grandis*)) and Russian sunflower husk (*Helianthus annus* L.) pellets were provided by EDF Energy plc (London, UK). South African eucalyptus (*Eucalyptus grandis*) pellets, miscanthus (*Miscanthus* x *giganteus*) pellets and American steam exploded white wood chip pellets were provided by E.ON UK plc (Coventry, UK). Microwave torrefied white wood pellets were sourced from a commercial supplier [42]. No information was available on the processing history of the pellets.

### 2.2. Pellet Mechanical Durability

Pellet mechanical durability of the biomass pellets was evaluated in accordance with BS EN ISO 17831-1 [8]. A test portion (*m_E_*) of 500 ± 0.1 g was placed in a tumbling box device, and then tumbled at 50 ± 2 RPM for 500 rotations. The sample was then passed manually through a 3.15 mm diameter sieve suitable for manual screening in accordance with BS ISO 3310-2:2013 [43]. After sieving, the sample remaining on the sieve was weighed (*m_A_*). The test was performed in duplicate. The mechanical durability of pellets (*D_U_*) was calculated as follows:

(1) $$D_{U}=\frac{m_{A}}{m_{E}}\times 100$$

### 2.3. Biomass Milling Energy

The pellets were subjected to a range of milling trails in several different types of laboratory scale mills [44]. Three different mills were used as part of this study: a Hardgrove mill, knife mill, and ring-roller mill. The mills were off-the-shelf unmodified laboratory mills, except for the ring-roller mill, which was a bespoke one-off laboratory test mill. Details of the milling tests can be found in previous publications by the authors [21,44]. The microwave pellets were not tested for Hardgrove Grindability Index (HGI) and durability, as they were not available at the time of testing. The statistical significance of the correlations between milling energy, durability, and mechanical strength tests were obtained in Minitab 17.1 (State College, PA, USA). The particle size range of the biomass particles (prior to densification) was obtained using a particle disintegration test (BS EN 16126:2012) [45], and details of this method can be found elsewhere [44]. Particle size distributions were determined by sieving the milled product in accordance with BS EN 15149-2:2010 [44,46].

### 2.4. Quasi-Static Mechanical Strength of Pellets

**Figure 1:**
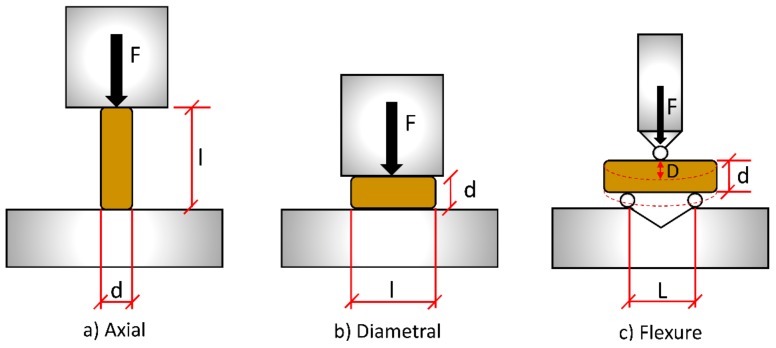
Axial (**a**), diametral (**b**), and flexure (**c**) mechanical test configurations.

Quasi-static mechanical compression tests were used to ascertain the mechanical strength of the pellets for low strain rates. The biomass pellet diameters and lengths were measured according to BS EN ISO 17829:2015 [47], and conformed with the requirements of BS EN ISO 17225-2/6 [9,10]. The biomass pellet diameters were 8.4 ± 0.2 mm for mixed wood pellets, 8.4 ± 0.1 mm for eucalyptus pellets, 8.6 ± 0.4 mm for sunflower pellets, 6.3 ± 0.2 mm for miscanthus pellets, 5.9 ± 0.1 mm for steam exploded pellets, and 5.8 ± 0.1 mm for microwave pellets. The biomass pellet lengths were 21.1 ± 7.0 mm for mixed wood pellets, 17.4 ± 4.8 mm for eucalyptus pellets, 14.2 ± 4.3 mm for sunflower pellets, 19.7 ± 4.8 mm for miscanthus pellets, 18.2 ± 5.6 mm for steam exploded pellets, and 22.8 ± 5.5 mm for microwave pellets. For the three-point bend test, samples with a length greater than the span, *L* (10.2 mm), were used. The pellets were tested in three orientations; axial, diametral, and flexure (Figure 1), on an Instron Mechanical 5969 testing system (Norwood, MA, USA). The strain rate was 1 mm/min with a 5 kN load cell for all tests, and the samples were tested in a similar approach to that adopted by Graham et al. [5,6]. For the axial tests, the ends of each pellet were first ground square using a bench grinder or sand paper. For the diametral compression tests, the pellets were placed on their sides and compressed laterally, while a three-point loading rig was used for the flexure tests. Ten pellets of each sample were tested in each orientation.

Several parameters were obtained from the test results. The proportional limit is defined as the highest point at which stress is directly proportional to strain (elastic limit), and is characterised by the proportional stress (*σ_p_*) and elastic limit (*ɛ_p_*). The ultimate strength (*σ_s_*) of the pellet is defined as the stress at the point of failure, and ductility (*ɛ_s_*) as the strain of the material at failure. The Young’s modulus (*E*) of the pellets was obtained from the gradient of the linear portion of the stress–strain curve. The compressive axial stress (*σ_a_*) was found by using the following formula:

(2) $$\sigma_{a}=\frac{F}{\pi r^{2}}$$

where *F* is the applied force (kN) and *r* is the radius of the pellet (mm). The compressive axial strain *ε_a_* was found by the following:

(3) $$\varepsilon_{a}=\frac{l-l_{i}}{l}=\frac{\Delta l}{l}$$

where *l_i_* is the compressed length (mm) of the pellet. The compressive diametral stress *σ_d_* was found from the following:

(4) $$\sigma_{d}=\frac{F}{rl}$$

where *l* is the length of the pellet (mm), and there is an assumed contact area of 50% of the diameter (*d*) or radius (*r*). The compressive diametral strain *ε_b_* was found as follows:

(5) $$\varepsilon_{d}=\frac{d-d_{i}}{d}=\frac{\Delta d}{d}$$

where *d_i_* is the diameter (mm) of the pellet after compression. For wood specimens, the modulus of rupture (*σ_f_*, denoted as flexure stress) reflects the maximum load-carrying capacity of a member in bending and is proportional to the maximum moment borne by the specimen. Flexure stress is an accepted criterion of wood strength, although it is not a true stress as the formula by which it is computed is valid only to the elastic limit [48], and gives the overall strength of a pellet. For the three-point bend test, the flexure stress *σ_f_* was found as follows:

(6) $$\sigma_{f}=\frac{FL}{\pi r^{3}}$$

where *L* is the distance between the support beams. The Flexure strain *ɛ_f_* was obtained as follows:

(7) $$\varepsilon_{f}=\frac{6Dd}{L^{2}}$$

where *D* is the deflection of the pellet under compression (mm).

### 2.5. Dynamic Mechanical Strength of Pellets—Split Hopkinson Bar

**Figure 2:**
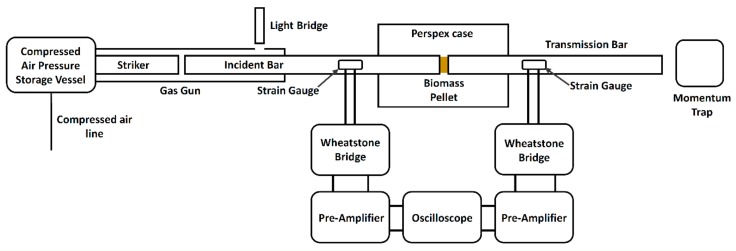
Split Hopkinson pressure bar (SHPB) schematic arrangement.

The Split Hopkinson pressure bar, also known as a Kolsky bar, is a dynamic stress–strain response characterisation tool for materials deforming at high strain rates (10^2^ to 10^4^ s^−1^) [36]. A SPHB consists of three major components, as shown in Figure 2; a loading device, bar components, and a data acquisition and recording system. The incident and transmission bars were acrylic bars with a Young’s modulus of 28,000 MPa, the mass of 0.65 kg, diameter of 25 mm, and length of 0.97 m. The striker was launched via a gas gun of compressed air in a pressure storage vessel into a long gun barrel, where it accelerated until it impacted the incident bar at constant speeds. The striking velocities were measured using a light bridge and were set by setting the pressure to 0.5 bar above ambient for all tests.

The sample to be tested was placed between the incident and transmission bar with a small amount of lubricant to hold the sample in place during the test. All samples had dimensions within the range used in the quasi-static tests (Section 2.4). Data were collected via strain gauges attached to the incident and transmission bar. The strain gauges used were Tokyo Sokki Kenkyujo Co. Ltd. FLA-6-11-3L strain gauges (Tokyo, Japan) with a gauge factor of 2.12% ± 1%. One strain gauge was located on the incident bar, and one was located on the transmission bar. The signals from the strain gauges are conditioned via a quarter Wheatstone bridge with an internal dummy resistor. The signal is amplified using a signal amplifier, which is then recorded on a computer using the AD-logger data acquisition software (version 1.13, ADLINK, New Taipei, Taiwan) with a correction factor of 0.21 from the oscilloscope calibration. The sampling frequency of the data acquisition software was 2 MHz. The quarter Wheatstone bridge has an excitation voltage *V_ex_* of 2 V, and the signal amplifier has an amplification factor (*AF*) of 100. The strain *ε* is calculated as follows:

(8) $$\varepsilon =\frac{4V_{out}}{V_{ex}GF\;AF}$$

where *V_out_* is the voltage measured by the strain gauge and *GF* is the gauge factor.

During the experiment, a stress wave is generated by the impact of the striker on the incident bar. These reflections build up the stress level in the specimen and compress the specimen. Part of the unloading wave is reflected back, and the rest transmits into the transition bar at the bar/specimen interface, while the specimen is unloaded. The incident and reflected pulses are measured by the strain gauge in the incident bar, and the transmitted pulse is measured by the strain gauges on the transmission bar. When stress waves propagate in a long rod, the mechanical energy of the stress wave takes the form of the strain energy though bar deformation and the kinetic energy of the bar motion. The average engineering strain rate ($\dot{e_{s}}$) in the specimen can be found as follows [36]:

(9) $$\dot{e_{s}}\left(t\right)=-2\frac{C_{b}}{L_{s}}\varepsilon_{R}\left(t\right)$$

where *L_s_* is the specimen length, *ɛ_R_* is strain in the reflected pulse (measured by a strain gauges), and *C_b_* is the elastic bar wave speed, which can be found from the following [36]:

(10) $$C_{b}=\sqrt{\frac{E_{b}}{\rho_{b}}}$$

where *E_b_* is the Young’s modulus of the bar and $\rho_{b}$ is the density of the bar. The average engineering strain (*e_s_*) in the specimen can be found by the following [36]:

(11) $$e_{s}\left(t\right)=2\frac{C_{b}}{L_{s}}\int_{0}^{t}\varepsilon_{R}dt$$

where *t* is the duration of the reflected pulse. The engineering stress ($s_{s}$) in the specimen can be found by the following [36]:

(12) $$s_{s}\left(t\right)=\frac{A_{B}}{A_{s}}E_{B}\varepsilon_{T}\left(t\right)$$

where the strain in the transmitted pulse is *ɛ_T_* (measured by strain gauges), *A_S_* is the cross-sectional area of the specimen, and *A_B_* is the cross-sectional area of the bar. From the equations for *e_s_* and *s_s_*, a stress-strain curve is obtained at a strain rate defined by an average taken over $\dot{e_{s}}$. Pellets were tested in axial and diametral orientations in the SHPB test and 10 repeats were conducted per sample. The data were collected and then processed in Matlab^®^ 2015a. The strain signal was differentiated to analyse the rate of change and inflection of strain rates in the experiment. Signal smoothing was conducted using the smoothn.m script by Garcia [49], which is an automated smoothing procedure for uniformly sampled data sets based on a penalised least squared method, which allows smoothing of the results in one or higher dimensions by means of the discrete cosine function. The results are presented as engineering strain, strain rate, and stress.

## 3. Results and Discussion

### 3.1. Pellet Durability

**Figure 3:**
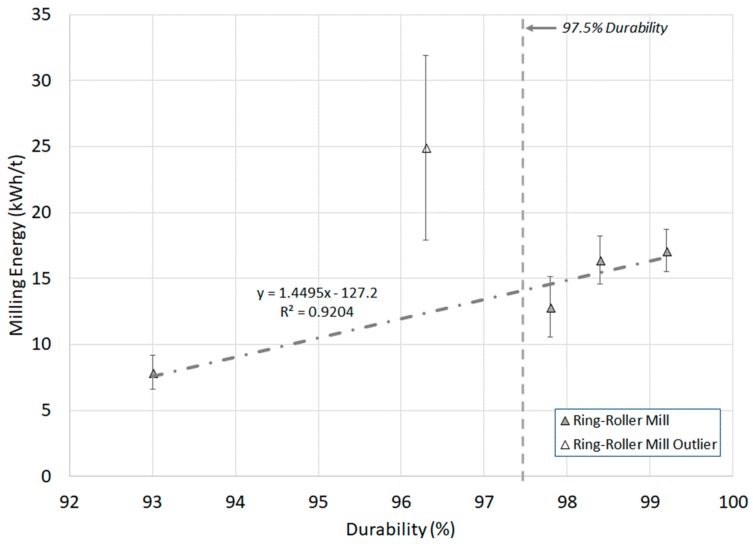
Ring-roller mill energy versus pellet durability. The acceptable limits of durability according to BS EN ISO 17225-2/6 for wood and non-wood pellets are noted. (Milling *n* = 3, durability *n* = 2).

**Figure 4:**
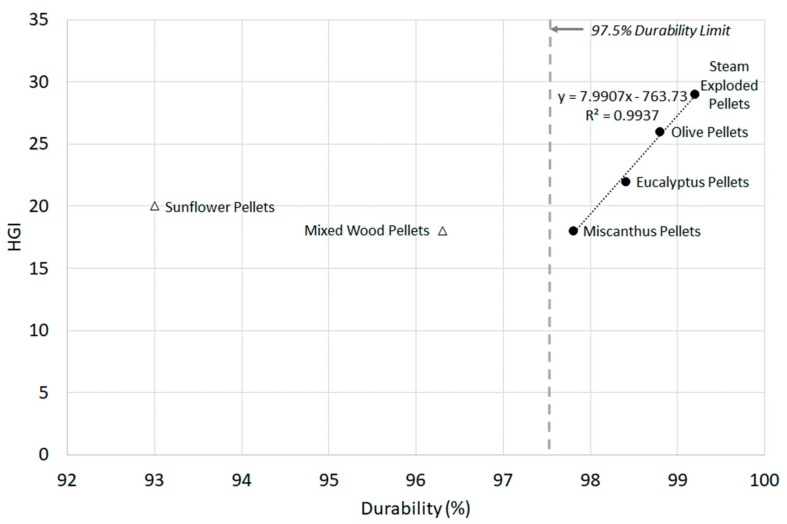
Pellet Hardgrove Grindability Index (HGI) values versus pellet durability. The acceptable limits of durability according to BS EN ISO 17225-2/6 for wood and non-wood pellets are noted (*n* = 2).

Mechanical durability is the standard ISO test for assessing the resistance of densified fuels towards shocks and/or abrasion because of handling and transportation processes. Unlike the quasi-static and dynamic mechanical strength tests, which are single pellet tests, it is a bulk material test. Apart from sunflower and mixed wood pellets, all samples conform with both ISO pellet specifications of 97.5% durability [9,10]. Figure 3 shows that a correlation exists between the ring-roller mill energy and pellet durability, which was previously noted in a knife mill [21]. Mixed wood pellets were an outlier for both tests because of mill choking [44]. Both mills showed a good fit (R^2^ > 0.92, R^2^ > 0.995 [21]), but more testing is required for other biomass varieties to validate the result. These correlations have not been noted previously in literature. Figure 4 shows that this correlation also exists for HGI (R^2^ > 0.99), but only for pellets with a durability over 97.5%. This suggests that low durability will not only result in a pellet that is unsuitable for transport, but will also have poor comminution behaviour. Thus, mechanical durability can provide an indication of how well a pellet will break down in a mill, as well as its suitability for transportation and handling.

Little was known about the densification process used to manufacture the pellets in this study beyond the pre-densified particle size distribution (Table 1) [44]. The microwave pellets did not disintegrate in the pellet disintegration test, and thus their pre-densified particle size distribution is unknown. Mixed wood and sunflower pellets had the largest particle size of all the pellets, with pre-densified 80th percentile particle size (*d*_80_) of 1373 and 1744 µm, respectively. Based on the durability and milling results (Figure 3 and Figure 4), it can be deduced that these pellets are composed of particles that are not only too large to form a durable pellet, but are also too large to be classified in mills. The recommended particle size for good pellet quality is 0.6 to 0.8 mm [50], with Franke and Ray [51] recommending a particle size of 0.5 to 0.7 mm for more durable pellets. Particles greater than 1 mm will act as predetermined breaking points in pellets, but the increased cost of production of fine pellets results in pellets with average particle sizes in excess of 1 mm. Additionally, the present study found no correlation between pellet mechanical strength and durability, which was previously noted by Larsson et al. [34].

### 3.2. Quasi-Static Mechanical Strength of Pellet

**Figure 5:**
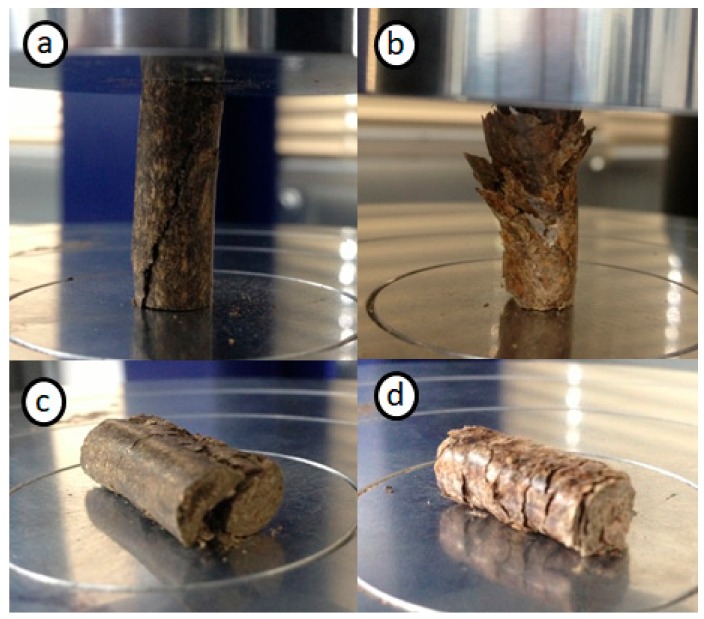
(**a**) Axial failure by shear (sunflower pellets), (**b**) axial failure by delamination (mixed wood pellets), (**c**) diametral failure by shear (sunflower pellets), and (**d**) diametral failure by delamination (mixed wood pellets).

The quasi-static compressive mechanical strength of commercially sourced biomass pellets has only been explored in a few studies, and results generally show a large variation due to the heterogeneous nature of biomass pellet composition [5,6]. In this study, two main failure modes were observed during the axial and diametral compression testing. Shear failure (Figure 5a,c) was observed for sunflower pellets, and steam exploded and microwave pellets. Secondly, delamination (Figure 5b,d) was observed for mixed wood and eucalyptus pellets. Miscanthus pellets showed both failure modes, especially in diametral compression tests where a delamination would result in a shear failure across the cross-section of the pellet. The failure mode observed during the quasi-static test was mainly that of ductile materials, with a progressive loading and gradual collapse (Supplementary Figure S1). Only steam exploded pellets showed a more brittle fracture mode, with the sudden collapse in axial and flexure modes. Microwave pellets also experience a more brittle fracture during the flexure test. Biomass pellet bonding relies on the strength of the bonds between the particles, and not the particles themselves. Pellet bonds are influenced by pellet processing methods, moisture content, and the quantity of extractives such as fatty acids and waxes [16]. Higher extractive contents has been shown to reduce pellet strength, as these substances form on the wood particle surface and prevent bonding between particles [24]. Delamination of the particles during the compression tests indicates that the bonding between the particle surfaces is weak, which could be linked to the extractive contents of the raw biomass.

The quasi-static mechanical properties of the pellets are summarised in Table 2. Proportional stress, elastic strain, and the Young’s modulus describe the properties up to their elastic limit (straight line) on the stress–strain curves, while ultimate stress and ductility describe the properties of the pellets at the point of failure. Ten pellets were used in each test, but the high standard deviation in Table 2 suggests that more runs are required to increase the accuracy of the test. Up to the elastic limit, the proportional stress of the samples does not vary significantly between orientations. Steam exploded pellets has identical proportional stress values in diametral and axial orientations (16.7 MPa), and only slightly lower in flexure (16.0 MPa), and similar trends were observed for miscanthus and sunflower pellets. However, microwave, eucalyptus, and mixed wood pellets showed diametral proportional stress that was almost twice the stress observed in axial and flexure orientations. 

The elastic strain was lowest for axially orientated pellets and highest for flexure. This resulted in high Young’s modulus values in the axial orientation, especially for steam-exploded pellets (871 MPa) compared with diametral (468 MPa) and flexure (278 MPa) orientations. The Young’s modulus represents a materials stiffness, and these results show that pellets show the highest rigidity in axial orientations under a uniformly distributed load, and poor rigidity when subjected to point loads (flexure). This is important for different processing conditions, as during storage and transport, loads will be uniformly distributed in axial and diametral orientations, but milling will involve more point loads. Thus, it is desirable to have higher Young’s modulus in axial and diametral orientations compared with flexure. Table 2 shows that at the point of failure, ultimate stress has not increased significantly beyond the proportional limit, but ductility (or strain at failure) has significantly increased. This suggests that the pellets are sustaining the load but collapsing before failure. In comparison to wood beams, biomass pellets have much lower values of Young’s modulus [48]. This is to be expected, as unlike timber, biomass pellets are heterogeneous particulate composites with no inherent structural components [52]. The strength of wood pellets made from untreated biomass arises from the thermal softening of lignin during the pelleting process [7]. This thermal softening of lignin polymer chains results in strong bonds upon cooling. The optimization of the pelleting process is essential to ensure that the strength of biomass pellets is such that pellets are durable during transport, but also relatively easy to disintegrate during milling.

### 3.3. Correlations between Quasi-Static Mechanical Strength and Milling Energy

**Figure 6:**
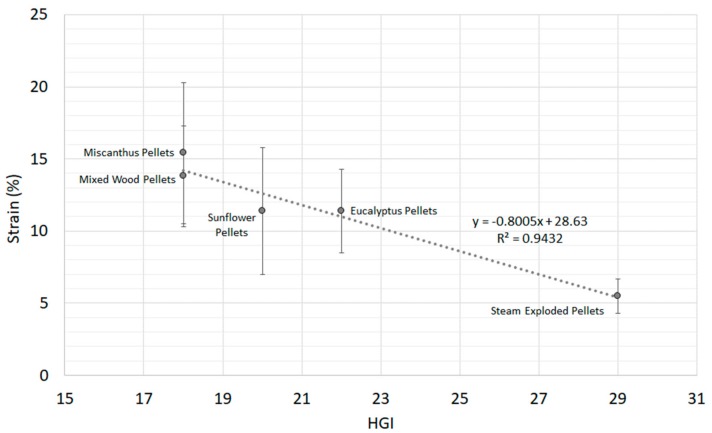
Biomass pellet diametral ductility versus HGI (HGI *n* = 2, ductility *n* = 10).

In order to evaluate any potential relationships between the quasi-static mechanical properties of the biomass pellets and their previously obtained milling energies [44], correlations were explored for all parameters using Minitab 17.1. Only correlations with *p*-values below 0.05 were considered statistically significant. Based on this, no correlations were observed for axial or flexure mechanical properties and milling energy. However, several correlations were observed for diametral compressive mechanical properties. The first correlation was between biomass strain and HGI (Figure 6). With a *p*-value of 0.006, the relationship was statistically significant. The correlation between diametral compressive ductility (strain at failure) versus HGI (Figure 6) shows a strong negative correlation (−0.97) with no unusual residuals, and a strong data fit (R^2^ > 0.94). A similar but weaker correlation was also observed between diametral compressive elastic strain and HGI (R^2^ > 0.82), and is shown in Supplementary Figure S2. The results suggest that ductility relates better to HGI for all pellet types than durability does, which only showed a correlation for samples with a durability above 97.5% (Figure 4).

**Figure 7:**
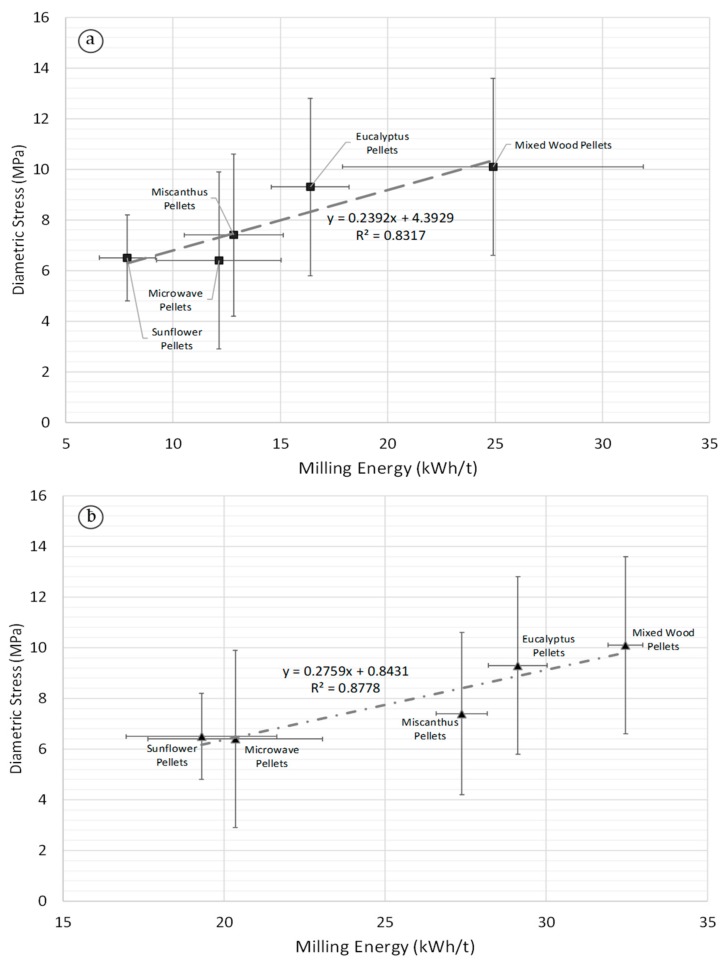
Ring-Roller (**a**) and knife mill (**b**) energy against biomass pellet diametral ultimate strength (milling energy *n* = 3, stress data *n* = 10).

Figure 7 shows that correlations also exist between milling energy in a ring-roller mill (Figure 7a) and knife mill (Figure 7b) against diametral ultimate stress for the biomass pellets. Similar correlations were obtained for the milling energy and diametral proportional stress for the pellets (Supplementary Figure S3). For both correlations, the steam exploded pellets were an extreme outlier, with much higher diametral stress values than the other pellets, and thus are not indicated in Figure 7. Steam explosion uses the introduction and release of high-pressure steam in a reactor in short periods of time to rapidly expand biomass material. This induces significant physical, chemical, and structural changes in the biomass, as well as makes lignin more available for binding during densification [1]. Steam explosion breaks the lignin down into moderately reactive low-molecular weight products while retaining their basic structure. In contrast, torrefaction is the slow heating of biomass in an inert environment up to a maximum temperature of 300 °C [20]. Torrefaction improves binding during densification by increasing the number of available lignin sites, breaking down the hemi-cellulose matrix, and forming fatty unsaturated structures. The results of this study indicate that the steam explosion process has fundamentally altered the nature of the biomass pellet structure compared with other non-thermally treated pellets and microwave pellets. This indicates that different torrefaction methods impact pellet strength differently because of the varying impact of the thermal pre-treatment process on the composition of the pellets.

The positive correlation (0.91) between ring-roller mill energy and diametral ultimate strength had a *p*-value of 0.031, with R^2^ > 0.87. The same trends were noted for the knife mill and diametral ultimate strength correlations (0.94), with a *p*-value of 0.019 and R^2^ > 0.87. Figure 7 indicates that the diametral stress of a biomass pellet can be used as an indicator of how well that pellet will break down in the mill. It must be noted that the quasi-static mechanical test of biomass pellets does not provide an indication of biomass particle grindability However, as Figure 7a illustrates, there are significant error bars relating to biomass pellet mechanical strength, which has been observed in other studies [5]. Only a few studies to date have examined the mechanical properties of commercially produced biomass pellets [5,6,7,35]. Commercially produced pellets are more variable in size and composition compared with pellets produced in a single pellet die in a laboratory, and thus more variation in the results is to be expected. However, the benefit of the mechanical testing machine used in this study is that it is a standard piece of equipment in mechanical engineering laboratories and test facilities, and thus can be readily used to assess biomass pellet mechanical strength.

### 3.4. Dynamic Mechanical Strength of Biomass Pellets

**Figure 8:**
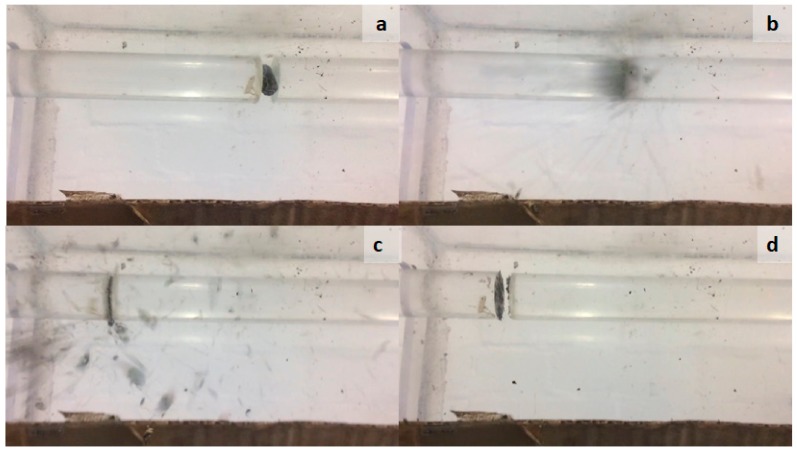
Video stills of SHPB test on mixed wood pellet in diametral orientation. Initial pellet position (**a**), initial impact (**b**), bars hitting the stop (**c**), final position of bars (**d**).

Quasi-static mechanical tests give the mechanical response of biomass pellets at low strain rates (10^−4^–10^0^ s^−1^) [22]. However, the actual strain rates experienced during milling are significantly higher. The Split Hopkinson pressure bar (SHPB) test provides a method of measuring the dynamic response of biomass pellets at high strain rates. Using a high speed camera (iPhone 5s, 120 frames per second), the dynamic response of a mixed wood pellets in the diametral orientation was recorded (Figure 8). Initially, the pellet was suspended between the incident and transmission bars (Figure 8a). Subsequently, the incident bar was hit by the striker and compressed the specimen into the transmission bar (Figure 8b). Both bars and the specimen travel horizontally until the transmission bar hits the stop and reverses direction, and then collides with the incident bar (Figure 8c). The energy transfer causes the transmission bar to accelerate away from the bar, resulting in the specimen material being released in an explosion-like manner. At the end of the test (Figure 8d), the pellet has completely disintegrated, and a small amount of pellet has been deposited on the contact faces of the bars.

**Figure 9:**
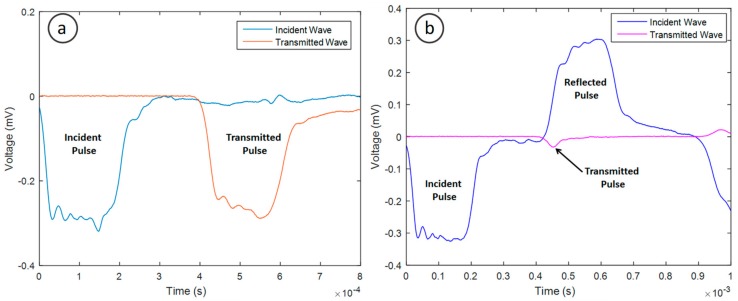
Incident and transmitted pulse for no sample condition (**a**) and incident, reflected and transmitted pulse for eucalyptus pellets in diametral orientation (**b**).

The impact results in a signal being recorded by the strain gauges on the incident and transmission bars. Figure 9a shows that for the no sample condition, virtually all the impact energy is transferred to the transmission bar upon impact. However, when a biomass pellet is placed between the two acrylic bars, the energy transferred is significantly reduced (Figure 9b). When the compression wave in the incident bar propagates to the interface between the incident bar and specimen, part of the wave is reflected back, and the rest is transmitted into the specimen. The wave is then reflected back and forth within the sample because of the mismatch of the wave impedance between the sample and bars.

The results of SHPB tests for the biomass pellets are presented in Table 3. The sample showed a higher Young’s modulus and compressive engineering stress in diametral orientation compared with the axial orientation. Unlike the results of Pierre et al. [53] for spruce wood, the samples did not compress but disintegrated upon impact, which is the desired effect for biomass pellets. However, as a constant strain rate condition was not achieved during the test, obtaining an accurate measurement of the pellet Young’s modulus was difficult. The maximum compressive stress did, however, occur within the area of constant strain rate, and thus can be taken as a measure of the strength of the materials. Steam exploded pellets showed the highest Young’s modulus and maximum compressive stress in axial and diametral orientations, and are thus the strongest of all the pellets. The other pellets had similar maximum compressive strengths in the diametral orientation (10 to 12 MPa) but varied in the axial orientation. None of the pellets experienced brittle failure (Supplementary Figure S4). Steam exploded, and microwave and miscanthus pellets sustained less than half the strain of mixed wood, eucalyptus, and sunflower pellets in the axial orientation, while in the diametral orientation, the microwave pellets had the highest strain of all the samples.

### 3.5. Correlations between Dynamic Mechanical Strength and Milling Energy

As with the quasi-static mechanical strength tests, correlations between the dynamic mechanical properties of the biomass pellets and their previously obtained milling energies [44] were obtained using Minitab 17.1. Unlike the quasi-static mechanical tests, there were no correlations between diametral mechanical properties and milling energy. Correlations were observed in the axial orientation however. The steam exploded, and eucalyptus pellets were outliers in all correlations, and thus were not included in regression analysis.

**Figure 10:**
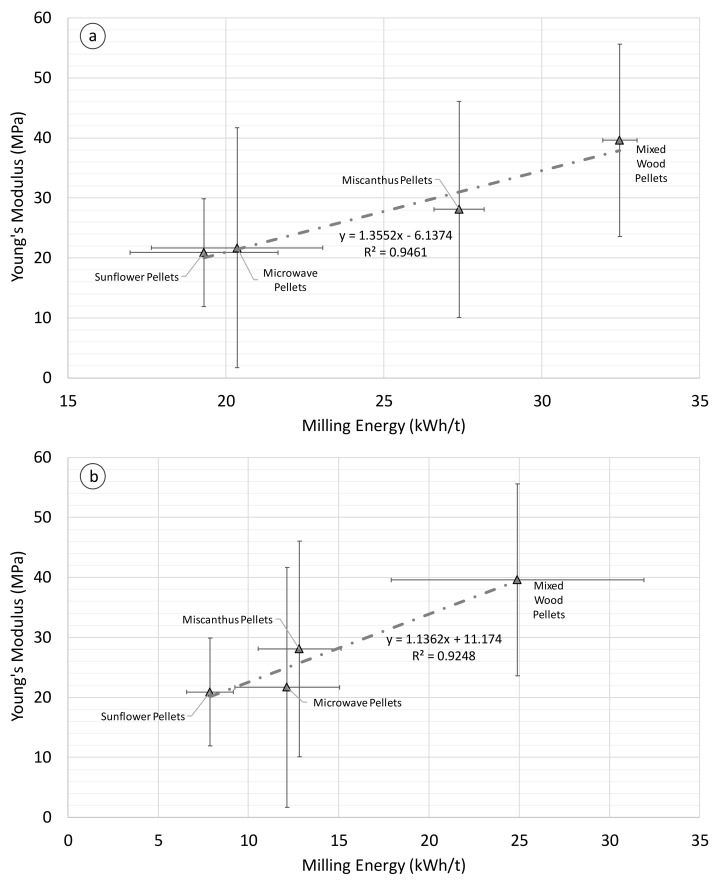
SHPB axial Young’s modulus against knife milling energy (**a**) and ring-roller (**b**) milling energy for biomass pellets (milling energy *n* = 3, *E_s_* data *n* = 10).

Correlations were obtained between the dynamic axial Young’s modulus and ring-roller and knife milling energies. For axial dynamic Young’s modulus and ring-roller milling energy (Figure 10a), the positive correlation (0.96) was statistically significant (*p* = 0.038) with a good fit (R^2^ > 0.92). The correlation was slightly stronger for axial dynamic Young’s modulus and knife milling energy (Figure 10b), with a positive correlation (0.97) and a *p*-value of 0.027, and a good data fit (R^2^ > 0.94). However, as Figure 10 illustrates, there is a large degree of uncertainty to the dynamic strength results. The poor repeatability of single pellet mechanical tests has been noted in previous studies [23], and thus further work is required to enhance the accuracy of the results through refined testing methodologies, such as the influence on pellet length and diameter on pellet strength. Despite this, the results indicate that correlations can be drawn between milling energy and dynamic strength tests.

### 3.6. Comparison of Quasi-Static and Dynamic Mechanical Strength

This study is the first to assess the dynamic mechanical strength of biomass pellets and relate it to milling energy. The results in Table 2 and Table 3 indicate that for some orientations, quasi-static and dynamic mechanical strength tests will provide similar mechanical strengths, while in other pellets it will vary significantly. In general, compressive stress was highest in the diametral orientation at low and high strain rates, which has also been observed for other cellulosic materials tested in an SHPB [40]. Brittle fracture was only observed in the flexure test for the steam exploded and microwave pellets (Figure S1). All other samples exhibited more ductile fracture (Figures S1 and S4).

For samples tested in the diametral orientation, pellet strength was greater at high strain rates. The greatest variation was observed for sunflower pellets, with the diametral quasi-static stress being 52% lower than the dynamic diametral stress. Interestingly, microwave pellets strength at low strain rates was 46% lower than at high strain rates, but steam exploded pellets were only 13% lower. The steam exploded pellets effectively maintained their strength across the strain range. This suggests that different thermal pre-treatments affect mechanical strength differently at low and high strain rates. Eucalyptus, mixed wood, sunflower, and microwave pellets had similar diametral Young’s modulus values at low and high strain rates. Only miscanthus and steam exploded pellets showed significant variances between high and low strain rates, with miscanthus’ quasi-static diametral Young’s modulus being 56% lower than its dynamic value, and 34% lower for steam exploded pellets. This indicates that these pellets are more rigid at higher strain rates. In the axial orientation, the overall pellet strength is greater at high strain rates in axial orientations, apart for steam exploded pellets (15% higher in the quasi-static test). The quasi-static stress was 10%–20% lower on average than the dynamic, apart from mixed wood, which was 47% lower. Further work is currently ongoing into the relationship between quasi-static and dynamic mechanical strength to pellet particle surface area, pellet breakage function, and milling energy.

The axial Young’s modulus showed a very different trend to all other parameters. The dynamic axial Young’s modulus was much lower in comparison with the quasi-static test, with reductions of 84% for microwave pellets, 83% for eucalyptus pellets, and 66% for mixed wood. Thus, the pellets are less rigid at high strain rates than at low strain rates in axial orientations. The variation in Young’s modulus in the axial orientation can be related to two issues. In the SHPB test, Young’s modulus is recorded in the non-linear strain rate region, whereas the strain rate is maintained at a constant level throughout the quasi-static test. This is a limitation of the dynamic test for soft materials, which have no structural integrity. As a result, they cannot withstand the impact long enough for failure to occur within the constant strain rate region. The second reason is because of the impedance mismatch with the bars and specimen. Conventional pressure bars have an impedance mismatch with soft materials because of the significant difference in longitude wave speed and density between the bars and the specimen [54]. This causes most of the wave to be reflected into the incident bar at the specimen bar interface. The stress wave transmitted through the sample has a very low amplitude, thus the signal to noise ratio can be of the same magnitude, making it impossible to record measurements. Further study is required into alternative bars with impedances closer to that of the biomass pellets to increase the signal strength and increase the accuracy of the readings.

## 4. Conclusions

This paper investigated the pellet durability and mechanical strength of commercially produced biomass pellets at high and low strain rates. Relationships between these mechanical properties and their milling energies in several mills were obtained. The paper presented the novel use of the Split Hopkinson pressure bar test to analyse dynamic mechanical response of biomass pellets at high strain rates. The pellet durability correlated with HGI (provided the pellet durability was over 97.5%) and the milling energies from a knife mill and ring-roller mill. The results indicate that low durability will result in pellets that are unsuitable for transport and comminution.

The compressive strength of the biomass pellets at low and high strain rates and in multiple orientations was obtained using quasi-static and dynamic mechanical tests. At low strain rates, the biomass pellets showed the highest rigidity in axial orientations under a uniformly distributed load (axial and diametral orientations), and poor rigidity when subjected to point loads (flexure test). This is desirable for storage, transport, and comminution processes. Dynamic mechanical strength and rigidity were highest in the diametral orientation. Pellet strength was greater at high strain rates than at low strain rates. Orientation was an important factor in relating mechanical strength to milling energy. Correlations were only observed in the diametral orientation at low strain rates and in the axial orientation at high strain rates. The results of the study indicated that different torrefaction methods have varying impacts on pellet strength. Mechanical tests of biomass pellets can provide an indication of how a pellet will break down in the mill, but further work is required to enhance their accuracy for commercially produced biomass pellets.

## Figures and Tables

**Table 1:** Pre-densified 80th percentile particle size (*d*_80_), durability, and milling energy data. HGI—Hardgrove Grindability Index.

Sample	Pre-Densified *d*_80_ (µm)	Durability (%)	HGI	Ring-Roller Energy (kWh/t)	Ring-Roller *d*_80_ (µm)	Knife Mill Energy (kWh/t)	Knife Mill *d*_80_ (µm)
Eucalyptus Pellets	1215	98	22	16.4 ± 1.8	958	29.1 ± 0.9	1171
Mixed Wood Pellets	1373	96	18	24.9 ± 7.0	1201	32.5 ± 0.5	1105
Miscanthus Pellets	1251	98	18	12.8 ± 2.3	1377	27.4 ± 0.8	1069
Sunflower Pellets	1744	93	20	7.9 ± 1.3	1523	19.3 ± 2.3	1145
Steam Exploded Pellets	1196	99	29	17.1 ± 1.6	521	21.8 ± 1.9	1412
Microwave Pellets	–	–	–	12.1 ± 1.9	538	20.4 ± 2.7	1091

**Table 2:** Mean and standard deviation (SD) of proportional stress limit *σ_P_*, elastic strain *ε_p_*, Young’s modulus *E*, ultimate strength (*σ_S_*), and ductility *ε_s_*, for biomass pellets tested in axial, flexure, and diametral orientations at low strain rates. Standard deviation (SD) is for 10 repeats of each sample.

Sample	Proportional Stress Limit *σ_P_* (MPa)		Elastic Strain *ε_p_* (%)		Young’s Modulus *E* (MPa)		Ultimate Strength *σ_S_* (MPa)		Ductility *ε_s_* (%)	
	Mean	SD	Mean	SD	Mean	SD	Mean	SD	Mean	SD
**Axial**										
Eucalyptus Pellets	4.5	1.7	5.6	2.2	87	39	5.5	1.8	10.3	3.0
Mixed Wood Pellets	3.9	0.8	3.4	0.6	115	24	4.1	0.8	5.4	2.0
Miscanthus Pellets	6.0	1.8	5.9	1.1	106	43	6.6	2.1	8.4	1.5
Sunflower Pellets	6.6	3.6	8.3	2.9	82	38	7.5	3.5	12.9	2.5
Microwave Pellets	3.1	0.8	2.3	0.5	140	48	4.0	0.8	5.2	1.9
Steam Exploded Pellets	16.7	4.4	2.1	0.6	871	331	19.8	3.9	3.1	0.8
**Diametral**										
Eucalyptus Pellets	7.7	3.1	5.7	1.6	133	35	9.9	3.5	12.0	2.9
Mixed Wood Pellets	7.9	2.9	6.5	1.5	120	25	9.5	3.5	13.8	3.5
Miscanthus Pellets	6.6	3.0	8.5	2.5	90	50	7.4	3.2	15.4	4.9
Sunflower Pellets	5.8	1.8	6.5	1.5	96	46	6.5	1.7	11.4	4.4
Microwave Pellets	5.5	3.1	7.3	2.7	73	19	6.4	3.5	11.2	4.4
Steam Exploded Pellets	16.7	2.8	3.6	2.8	468	71	18.4	3.2	5.5	1.2
**Flexure**										
Eucalyptus Pellets	2.2	0.9	16.6	2.9	15.4	6.8	2.4	1.0	19.2	4.4
Mixed Wood Pellets	2.3	1.3	16.9	3.4	15.2	7.5	2.7	1.3	26.0	10.1
Miscanthus Pellets	2.6	1.0	10.5	2.9	31.6	17.8	3.0	1.1	13.4	4.0
Sunflower Pellets	2.5	1.4	18.0	6.4	18.0	11.7	2.9	1.8	23.9	9.4
Microwave Pellets	3.1	1.7	7.4	2.4	40.9	15.7	3.4	1.8	8.8	3.3
Steam Exploded Pellets	16.0	3.8	6.7	1.2	277.9	56.3	16.4	3.9	6.9	1.1

**Table 3:** Mean diametral and axial compressive Young’s modulus (*E_e_*), engineering stress (*s_s_*), and engineering strain rates $\left({\dot{e}}_{s}\right)$.

Sample	$\mathrm{Diametral}\;{\dot{e}}_{s}\;(s^{-1})$	Diametral *s_s_* (MPa)	Diametral *E_e_* (MPa)	$\mathrm{Axial}\;{\dot{e}}_{s}\;(s^{-1})$	Axial *s_s_* (MPa)	Axial *E_e_* (MPa)
Eucalyptus	1257	11	129	1301	5	15
Mixed Wood	1130	11	124	1400	7	40
Miscanthus	679	12	205	719	7	28
Sunflower	1274	12	113	1283	8	21
Steam Exploded	1040	19	707	830	15	140
Microwave	1820	10	57	1329	3	22

## Supplementary Materials

The following are available online at http://www.mdpi.com/1996-1944/11/8/1329/s1, Figure S1: Typical quasi-static stress strain curves for biomass pellets in axial (a), diametric (b), and flexure (c) orientations. Figure S2: Biomass pellet quasi-static diametric elastic strain versus HGI (HGI *n* = 2, strain data *n* = 10). Figure S3: Ring-Roller (a) and knife mil (b) energy against biomass pellet quasi-static diametric proportional stress (milling energy *n* = 3, stress data *n* = 10). Figure S4: Typical dynamic stress strain curves for biomass pellets in axial (a) and, diametric (b), orientations.
