# Supplementary material for: Applicability of Mechanical Tests for Biomass Pellet Characterisation for Bioenergy Applications
Source: Materials (Basel). 2018 Jul 31;11(8):1329. doi: 10.3390/ma11081329 (PMC6119871; doi:10.3390/ma11081329)
Supplement: Supplementary file 1 [file materials-11-01329-s001.pdf]

materials

Supplementary Materials

Supplemental Data: Applicability of Mechanical Tests for Biomass Pellet Characterisation for Bioenergy Applications

Orla Williams *, Simon Taylor, Edward Lester, Sam Kingman, Donald Giddings and Carol Eastwick

Faculty of Engineering, the University of Nottingham, University Park, Nottingham NG7 2RD, UK; taylor_simon@hotmail.co.uk (S.T.); edward.lester@nottingham.ac.uk (E.L.); sam.kingman@nottingham.ac.uk (S.K.); donald.giddings@nottingham.ac.uk (D.G.); carol.eastwick@nottingham.ac.uk (C.E.)

* Correspondence: orla.williams@nottingham.ac.uk; Tel.: +44-1159515900

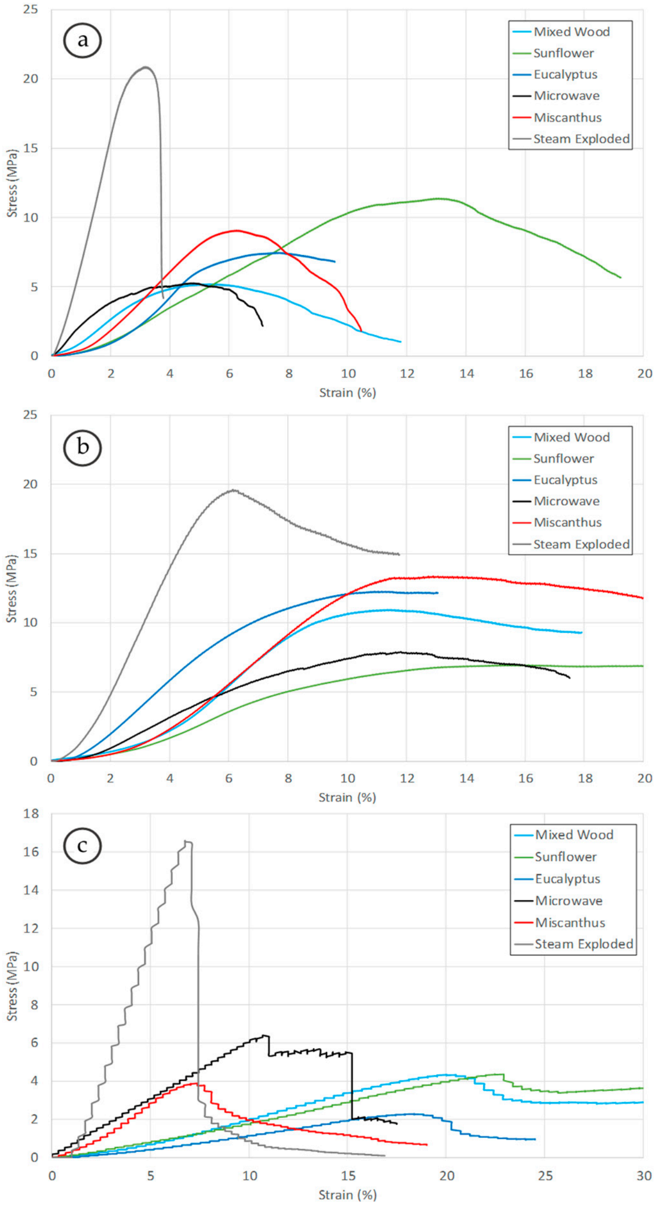

Figure S1. Typical quasi-static stress strain curves for biomass pellets in axial (**a**), diametric (**b**), and flexure (**c**) orientations.

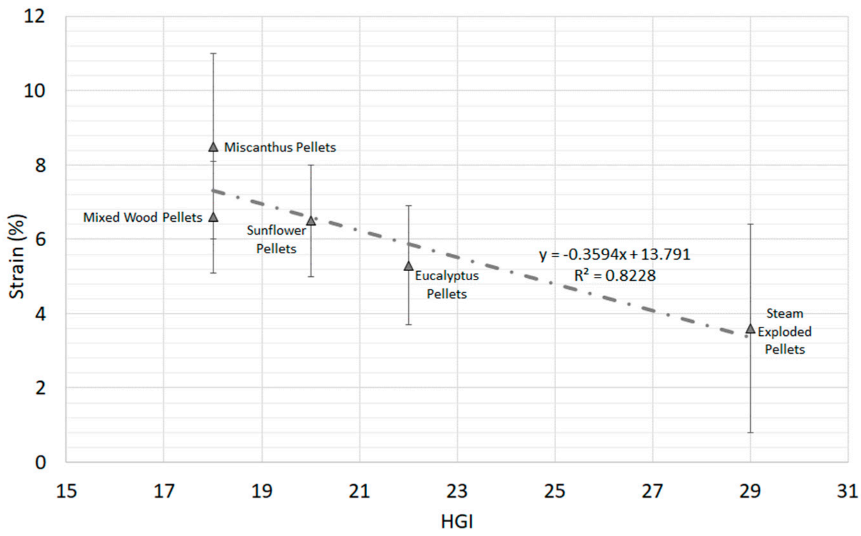

Figure S2. Biomass pellet quasi-static diametric elastic strain versus HGI (HGI n=2, strain data n=10).

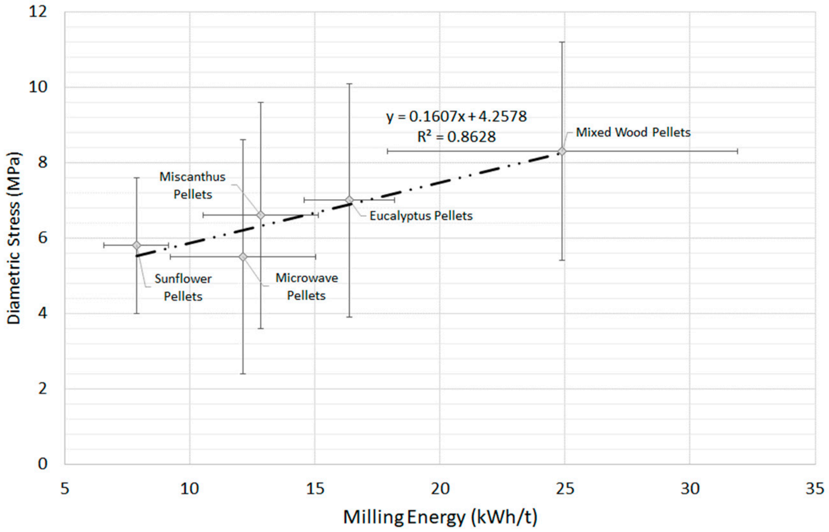

(**a**)

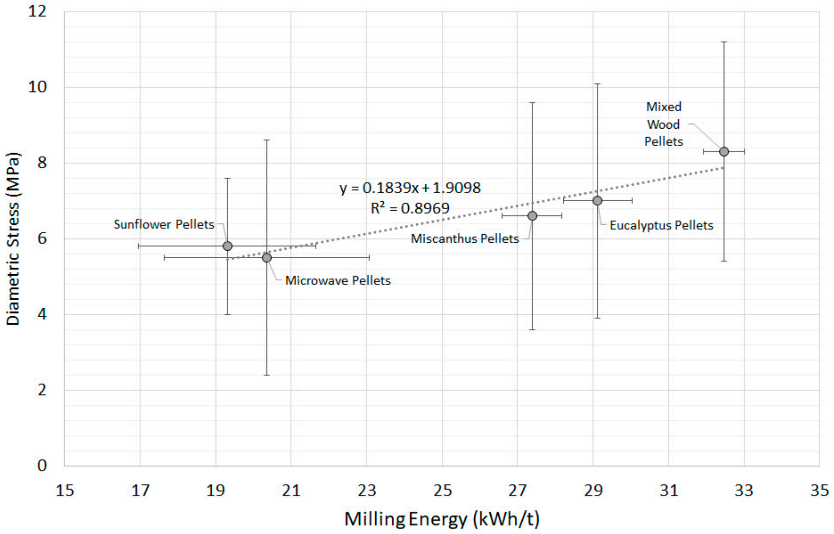

(**b**)

Figure S3. Ring-Roller (**a**) and knife mil (**b**) energy against biomass pellet quasi-static diametric proportional stress (milling energy N=3, stress data N=10).

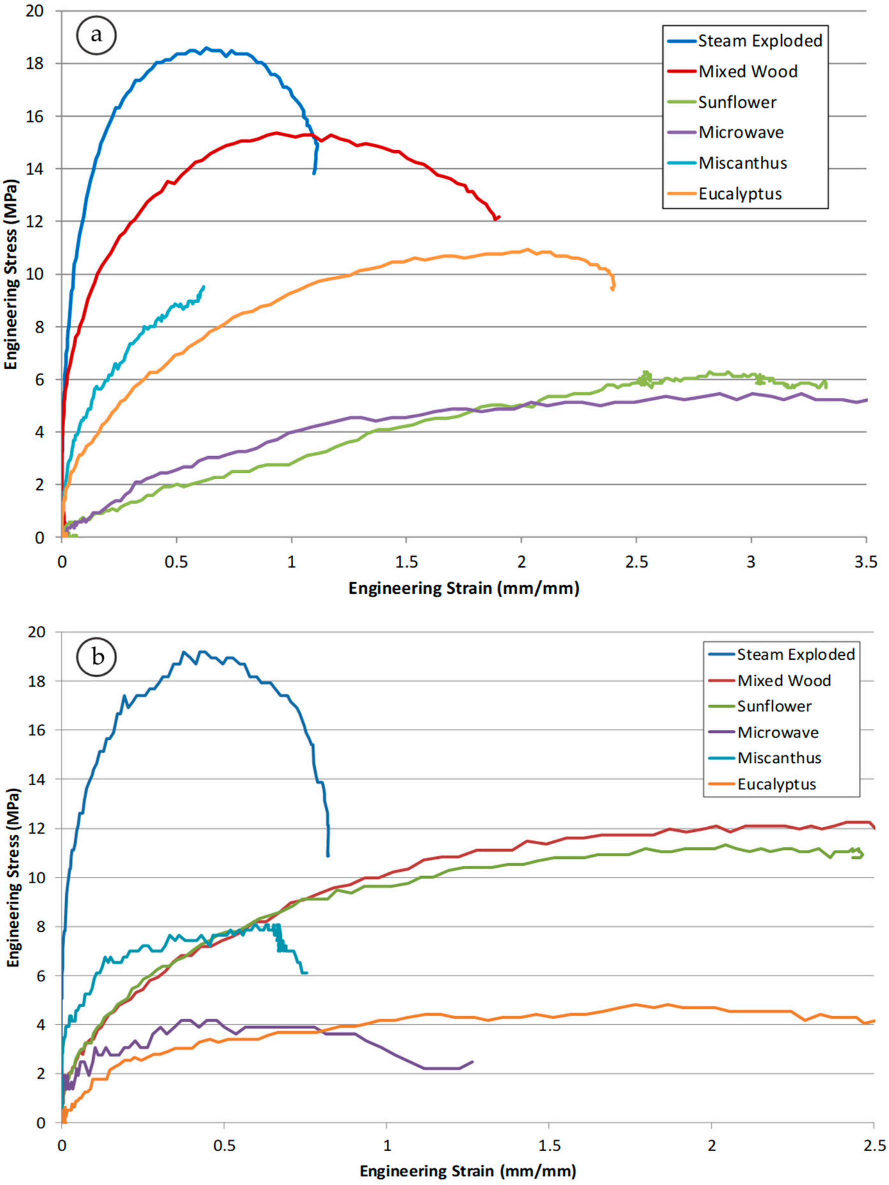

Figure S4. Typical dynamic stress strain curves for biomass pellets in axial (**a**) and, diametric (**b**), orientations.

© 2018 by the authors. Submitted for possible open access publication under the terms and conditions of the Creative Commons Attribution (CC BY) license (http://creativecommons.org/licenses/by/4.0/).
